# Structure and Location of Protein Sites Binding Self-Associated Congo Red Molecules with Intercalated Drugs as Compact Ligands—Theoretical Studies

**DOI:** 10.3390/biom11040501

**Published:** 2021-03-26

**Authors:** Ptak-Kaczor Magdalena, Kwiecińska Klaudia, Korchowiec Jacek, Chłopaś Katarzyna, Banach Mateusz, Roterman Irena, Jagusiak Anna

**Affiliations:** 1 Department of Bioinformatics and Telemedicine, Faculty of Medicine, Jagiellonian University Medical College, Medyczna 7, 30-688 Krakow, Poland; magdalena.ptak@uj.edu.pl (P.-K.M.); mateusz.banach@uj.edu.pl (B.M.); irena.roterman-konieczna@uj.edu.pl (R.I.); 2 Department of Theoretical Chemistry, Faculty of Chemistry, Jagiellonian University, K. Gumiński, Gronostajowa 2, 30-387 Kraków, Poland; klaudia.kwiecinska@student.uj.edu.pl (K.K.); korchow@chemia.uj.edu.pl (K.J.); 3 Faculty of Medicine, Chair of Medical Biochemistry, Jagiellonian University Medical College, Kopernika 7, 31-034 Krakow, Poland; kchlopas@su.krakow.pl

**Keywords:** drug carrier, albumin, hydrophobicity, hydrophobic core, doxorubicin, light chain of IgG, Bence-Jones protein, fuzzy oil drop model, albumin

## Abstract

In the search for new carriers capable of transporting toxic drugs to a target, particular attention has been devoted to supramolecular systems with a ribbon-like micellar structure of which Congo red is an example. A special promise of the possible use of such systems for directing drugs to a target emerges from their particular affinity to immune complexes and as an independent property, binding many organic compounds including drugs by intercalation. Serum albumin also appeared able to bind micellar particles of such systems. It may protect them against dilution in transport. The mathematical tool, which relies on analysis of the distribution of polarity and hydrophobicity in protein molecules (fuzzy oil drop model), has been used to find the location of binding area in albumin as well as anchorage site for Congo red in heated IgG light chain used as a model presenting immunoglobulin-like structures. Results confirm the suggested formerly binding site of Congo red in V domain of IgG light chain and indicated the cleft between pseudo-symmetric domains of albumin as the area of attachment for the dye.

## 1. Introduction

Therapies that rely on highly toxic drugs such as Doxorubicin (Dox), often applied in cancer treatment, are a double-edged sword. The drug does indeed preferentially destroy cancer cells due to their increased susceptibility caused by frequent division, but its deleterious influence on other tissues (particularly bone marrow) is also well understood, hence the concerns about its toxicity.

One possible solution to this dilemma would be to ensure that the drug acts only upon its intended target, limiting any potential side effects. Many attempts have been made to bring about such an outcome. One of them includes administering the drug in complex with a carrier, limiting its toxic effects and enabling rapid elimination of surplus drug molecules. Supramolecular systems—particularly those which form ribbonlike micelles (of which Congo red is an example)—are a promising lead in this respect [1].

Supramolecular Congo red micelles may incorporate many planar, mostly positively charged molecules including drugs (for example Dox) by intercalation. The further advantage in this respect is the selective attachment of Congo red to antibodies engaged in immune complexes but not to free antibody molecules. Among their major advantages is the ability to bind albumin as well as selective affinity for antibodies that form immune complexes. This property makes them a convenient carrier in targeted drug therapy [2].

Selective complexation of Congo red by antibodies engaged in immune complexes becomes possible due to structural modification of antibodies caused by internal tension, which emerges when an antibody interacts with an antigen. It opens the way to elaboration of a new immunotargeting technique. The problem is, however, that Congo red micellar structures may lose their cohesion and binding capability upon dilution in transport to the target. In this situation, albumin seems to come with help. Albumin binds the large micellar fragment of Congo red together with the intercalated drug micelles stabilizing it. Amyloids and many partly unfolded proteins, as for example IgG light chain studied mostly in this respect, may also incorporate self-assembled Congo red molecules, but albumin binding capacity is higher. In contrast to other protein molecules, it binds Congo red without the necessary structural modification [3].

The active site that binds Congo red is located in a gap between two pseudo-symmetrical fragments of albumin. The gap is also capable of binding fatty acids; however, its interaction with supramolecular dyes is unique and calls for a more in-depth structural analysis of the binding site, as the supramolecular ligand Congo red interacts with the protein in an atypical manner. To locate potential binding sites for the dye itself, structural studies have been carried out, based on the fuzzy oil drop model (FOD). The model makes it possible to determine the distribution of polarity/hydrophobicity throughout the protein and pinpoint likely binding sites ready to incorporate large ligands.

## 2. Materials and Methods

### 2.1. Data

The object of our analysis is the crystal structure of albumin and the IgG light chain, as listed in PDB for both proteins (Table 1) [4,5].

Program PyMol was used for 3D structure presentations [https://pymol.org/2/] (accessed on 25 January 2021). Charts were plotted using Matplotlib library [https://matplotlib.org/] (accessed on 28 January 2021).

### 2.2. Force Field

The structural analysis described below is based on the fuzzy oil drop model. As the model itself has been thoroughly described in numerous publications [6,7], we will restrict ourselves to a recapitulation of its core concepts, enabling interpretation of the presented results.

The status of the hydrophobic core in a protein molecule is described by the so-called Relative Distance (RD) parameter, which expresses the alignment between the actual distribution of hydrophobicity observed in the protein and an idealized (theoretical) distribution expressed by a 3D Gaussian. Good alignment between both distributions indicates the presence of a well-defined monocentric hydrophobic core, encapsulated by a hydrophilic shell. The degree of accordance is determined through quantitative comparison of differences between the theoretical (Gaussian) distribution (denoted T) and the observed distribution (denoted O), which depends on the location of amino acid residues in the protein as well as on their intrinsic hydrophobicity (according to any commonly accepted scale [6,7]). The latter distribution is computed using Levitt’s formula [8], while the quantitative difference between both distributions bases on the divergence entropy (DKL) function proposed by Kullback and Leibler [9]. As DKL is a measure of entropy, it cannot be interpreted on its own; for this purpose, another reference distribution (denoted R) is introduced, ascribing a hydrophobicity value of 1/N to each residue (where N is the number of residues in the protein).

Under these conditions, comparing the value of DKL for distributions O and T (O-T) with the corresponding value computed for distributions O and R (O-R) enables us to determine the “closeness” of O to each reference distribution. This comparison forms the basis for calculation of RD (given as O-T divided by the sum of O-T and O-R). When RD < 0.5, we conclude that the protein contains a hydrophobic core.

Assessment of proteins presented in this work will be performed on the basis of RD values calculated for entire complexes, individual chains, and specific domains treated as distinct structural units (with the 3D Gaussian adjusted appropriately in each case). 

When dealing with local discordances between T and O, surplus hydrophobicity suggests exposure of hydrophobic residues on the protein surface, which may indicate a ligand binding or protein complexation site [10], while a local deficit of hydrophobicity often corresponds to a binding pocket capable of accommodating a ligand (or substrate) [11].

In addition to hydrophobicity, the observed and theoretical distributions are also compared for electrostatic and van der Waals (dipole-dipole) interactions in order to discover local deviations for each interaction separately. This enables us to pinpoint active groups in protein molecules.

Our analysis aims to identify potential binding sites for a large supramolecular ligand, specifically, a Congo red micelle with intercalated Dox.

## 3. Results

Analysis of albumin and the IgG light chain, focusing on the structure of their respective hydrophobic cores, reveals the capacity for complexation of a large supramolecular ligand: a Congo red co-micelle with intercalated Dox. This section will be subdivided into two parts. The first part discusses albumin, both as a complete molecule and with regard to individual domains, while the second part will be devoted to the IgG light chain, with particular focus on its V domain.

### 3.1. Assessment of the Status of Domains Comprising Human Albumin in the Context of Their Capacity for Binding Congo Red in Complex with Dox Based on the Fuzzy Oil Drop Model

**Figure 1:**
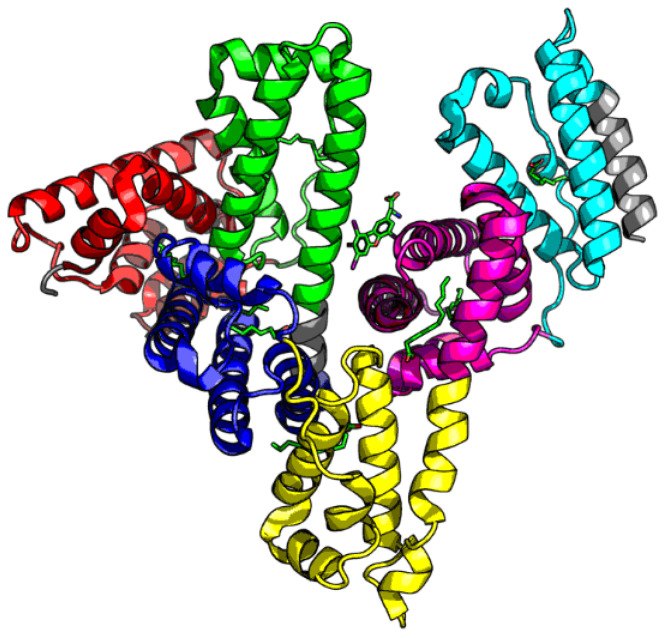
3D presentation of the albumin molecule. A—CATH domains (AI—red, AII—green, AIII —blue, BI—yellow, BII—magenta, BIII—cyan); green—disulfide bonds.

Assessment of the distribution of hydrophobicity in the complete human albumin molecule is provided in Table 2 along with Figure 1, which presents theoretical (idealized) and observed hydrophobicity distribution profiles (the latter resulting from inter-residual interactions). RD > 0.5 indicates discordance vs. the theoretical distribution, whereas RD < 0.5 suggests a coherent micellar structure.

Albumin is capable of binding fatty acids and many other individual molecules, including drugs. However, Congo red is a supramolecular ligand (as it is in form of Congo red-Dox co-micelle) whose interaction with albumin requires an in-depth explanation. Table 2 lists RD values calculated for various portions of the albumin molecule. When analyzed as individual structural units, three of six domains exhibit the presence of a monocentric hydrophobic core.

For albumin, an RD value of 0.741 is obtained, which indicates that no hydrophobic core (as defined by the fuzzy oil drop model, where distribution of hydrophobicity is modeled as a 3D Gaussian) exists in this protein. The aforementioned value should be regarded as high compared to other analyzed proteins. However, removal of residues for which the greatest discordance between T and O is observed, enables us to identify parts of the albumin chain that satisfy the requirements of the fuzzy oil drop model by representing micelle-like properties. The likely role of these parts is to stabilize the structure of albumin (given the common view that factors which contribute to structural stability include the presence of a hydrophobic core and disulfide bonds). In contrast, local discordances between T and O indicate the likely sites where interaction may occur.

**Figure 2:**
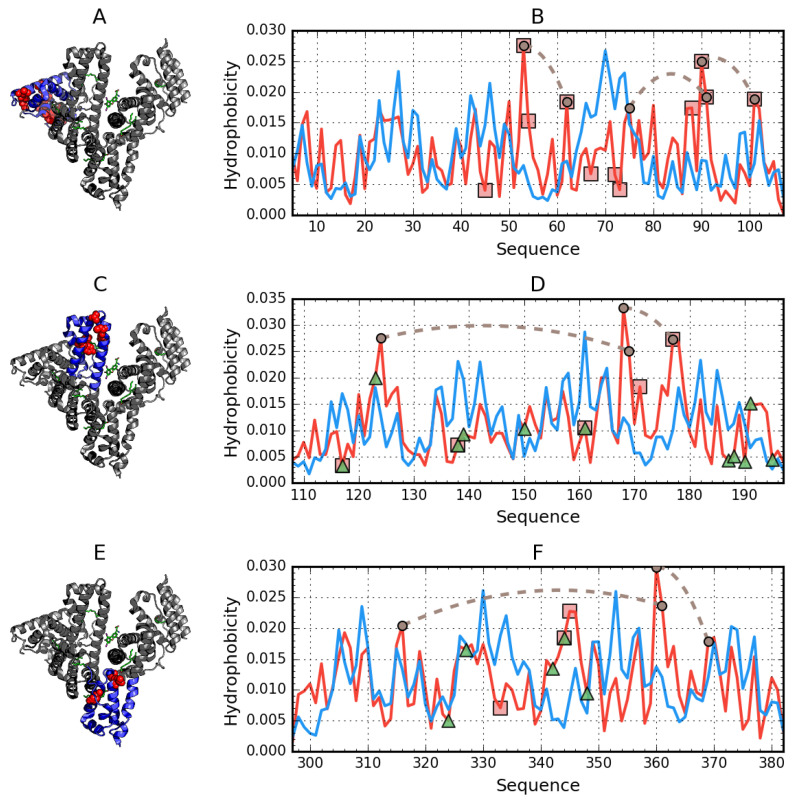
Hydrophobicity density distributions and 3D presentation of discordant domains in albumin: (**A**,**B**)—domain AI (5–107); (**C**,**D**)—domain AII (108–197); (**E**,**F**)—domain BI (297–382). Blue lines—theoretical (T) distribution; red lines—observed (O) distribution; brown dashed curves—disulfide bonds; square markers (and red spheres in the 3D view)—residues exhibiting discordance with the FOD model; triangle markers—ligand binding.

Figure 2 distinguishes parts of the protein for which micelle-like hydrophobicity distributions are observed. The status of domains treated as distinct structural units is significantly different than the status of the same domains treated as components of the overall structure of albumin. It should be noted that in order to analyze a domain as a standalone entity, the 3D Gaussian should be adapted so that it specifically encapsulates that domain, in contrast to a protein-wide 3D Gaussian, which enables us to determine the status of a given domain as part of the complete protein.

On the other hand, when treating domains as fragments of the larger albumin molecule, strong discordance is generally observed. This indicates that the resulting arrangement of domains does not produce a structure, which would contain a shared hydrophobic core. Discordant sites can be regarded as potentially capable of interaction with other molecules. Only domains AIII, BII, and BIII are somewhat aligned with the theoretical distribution when treated as individual structural units. As shown in Figure 1, when considering the molecule as a whole, discordance is observed for relatively long portions of the residue chain, whereas when treating domains as individual structural units, only selected residues remain discordant. In fact, in the latter case, three of six domains exhibit strong micelle-like conformational properties.

The distribution of nonbonding interactions (electrostatic and vdW) is likewise far from centric (Figure S1). In particular, electrostatic interactions appear rather evenly distributed across the structure of the protein Table S1.

Interpretation of results provided in Table 2 points to stabilization of domains AIII, BII, and BIII mediated by the presence of a well-defined hydrophobic core.

The status of fragments bracketed by disulfide bonds is also strongly consistent with the theoretical distribution (Table S2), with only one discordant fragment identified in each of the following domains: AII, BI, and BII. This suggests that the positioning of Cys residues in the process of forming disulfide bonds is consistent with the overall trend to produce a hydrophobic core.

Disulfide bonds are known to promote tertiary conformational stability; however, fragments bracketed by Cys residues for which high values of RD are obtained may be regarded as susceptible to structural rearrangements. Further analysis of the status of such fragments is provided in Table S2.

### 3.2. Ligand Binding by Albumin

Albumin is responsible for transporting certain hormones, drugs, fatty acids, and bile pigments. The ability to bind ligands is critical to its function and we can therefore expect its binding pockets to be well defined. The fuzzy oil drop model provides a way to analyze the properties of ligand-binding residues. The status of both loci (LIG and NO-LIG) with respect to the molecule as a whole is difficult to interpret given the structural complexity of albumin; however, when dealing with individual domains, ligand-binding residues may be pinpointed by looking for higher-than-average values of RD compared to the remainder of the protein chain. This is a consequence of the amino acids being arranged in a way that permits complexation of a specific ligand. According to the fuzzy oil drop model, local discordance indicates the specificity of a given location within the framework of its host structural unit. Thus, results obtained for albumin are consistent with the stipulations of the fuzzy oil drop model.

The fuzzy oil drop model can be used to determine the status of binding sites by comparing the observed hydrophobicity of ligand-binding residues with their corresponding theoretical hydrophobicity. Eliminating such residues from RD calculations carried out for individual domains (except for AI, where the ligand is not part of the structure listed in PDB–1HK4) causes a marked reduction in the value obtained for the whole structural unit. An interesting situation is observed for domain AIII, where ligand-binding residues belonging to the domain remain consistent with the model, while the remainder of the domain exhibits somewhat higher RD. Eliminating residues that comprise the binding site elevates the RD value for the remaining portion of the molecule Table S3.

The tertiary structure may also be analyzed by looking at the status of fragments which correspond to individual secondary folds. The presence of a discordant helix (or a beta fold, which, however, is not observed in albumin) also introduces specific local instability (Table S4).

Figure 2 provides a comparison of theoretical and observed distributions for domains treated as individual structural units for which RD remains greater than 0.5. Residues involved in ligand binding have been highlighted in the corresponding 3D structures.

**Figure 3:**
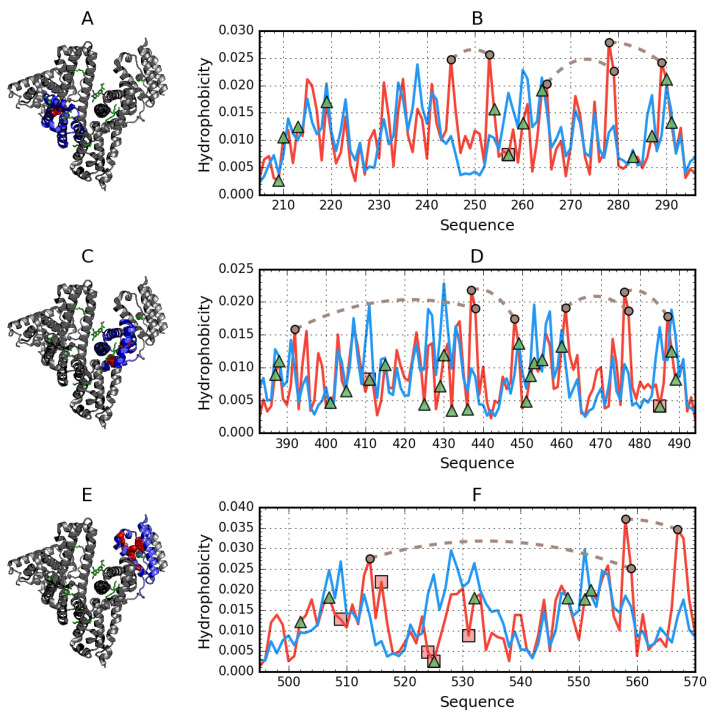
Hydrophobicity density distributions and 3D presentation of accordant domains in albumin: (**A**,**B**)—domain AIII (205–297); (**C**,**D**)—domain BII (383–494); (**E**,**F**)—domain BIII (495–570). Blue lines—theoretical (T) distribution; red lines—observed (O) distribution; brown dashed curves—disulfide bonds; square markers (and red spheres in the 3D view)—residues expressing discordance with the FOD model; triangle markers—ligand binding.

Figure 3 provides analogous information for domains for which RD remains below 0.5. Here, the highlighted residues exhibit deviations from the theoretical distribution in spite of the chain being generally accordant. These residues are involved in ligand binding, thus their misalignment with T may be interpreted as intentional and required to accommodate the ligand.

Table S3 reveals the curious status of domains BII and BIII, which represent uniform distribution of electrostatic and vdW interactions (effectively “opposite” to a monocentric distribution), but are otherwise well aligned with the monocentric model in terms of hydrophobic forces. This suggests that both domains (BII and BIII) are among the most stable in the analyzed protein. Notably, uniform distribution of electrostatic forces is regarded as appropriate for this type of interaction.

Binding a large ligand, such as the supramolecular structure discussed in this publication, does not appear to disrupt the structure of domains BII and BIII (as well as AIII). These domains are stable as a whole, while the remaining domains may undergo deformations leading to structural rearrangements. However, given the relatively large size of the ligand, the most likely site of complexation appears to be interdomain clefts, especially those which separate domains AII, BI, and BIII. The domain structure outlined in Figure 2 and Figure 3 illustrates the variations in the stability of various parts of the protein.

### 3.3. Albumin Molecule Analyzed as a Whole by Applying the Fuzzy Oil Drop Model 

#### Interpretation of Results Obtained for the Complete Structure of Albumin

**Figure 4:**
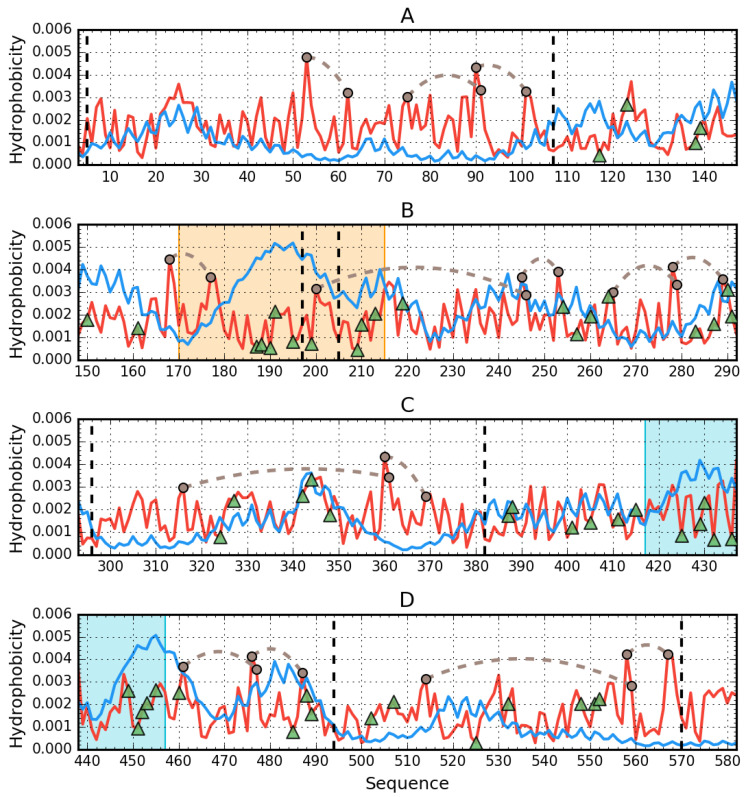
Hydrophobicity density distributions calculated for the whole albumin molecule, split into four subplots for clarity. Blue lines—theoretical (T) distribution; red lines—observed (O) distribution; brown dashed curves—disulfide bonds; vertical dashed lines—domain boundaries. Colored backgrounds mark the location of 175–215 (orange) and 417–467 (cyan) helices in the sequence. (**A**–**D**)—sequential fragments in chain. The blue line—theoretical distribution; expected distribution expressing the status of centric hydrophobic core, which is not present, since the O distribution observed reveals significant deficiency, suggesting a cleft.

The molecule as a whole is characterized by an RD value far in excess of 0.5, as shown in Figure 4. 

**Figure 5:**
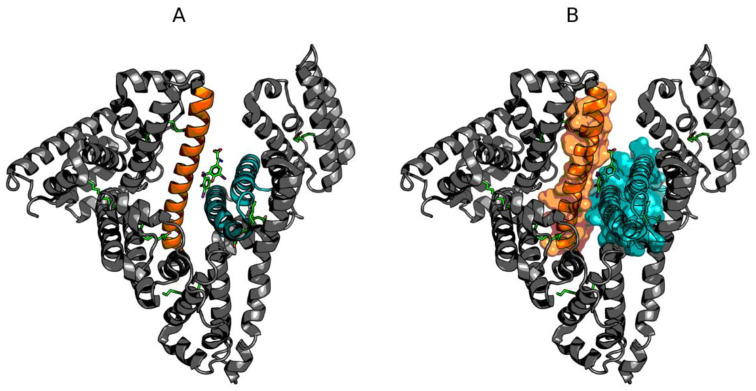
3D presentations of the albumin molecule (grey) with highlighted 175–215 (orange) and 417–467 (cyan) helices. (**A**)—without surface; (**B**)—with surface.

Figure 4 also reveals the presence of a highly discordant fragment (170–215). Given that this fragment does not belong to any of the domains, its status may be analyzed only in relation to the entire molecule. In the 3D structure, it corresponds to a centrally placed helix (Figure 5).

The helical fragment at 417–467 remains in direct contact with the central discordant helix. This fragment comprises two separate helices arranged into a hairpin. As shown in Figure 4, it is also strongly discordant.

**Figure 6:**
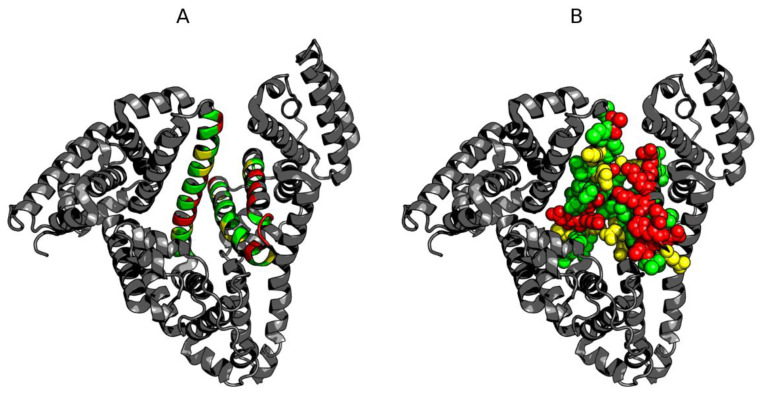
3D presentations of the albumin molecule (grey) with highlighted 175–215 and 417–467 helices. (**A**)—ribbon presentation; (**B**)—spatial presentation. Red—hydrophobic, yellow—hydrophilic—negative, green—polar.

The helical structures of polypeptides, which form the potential binding cavity for a supramolecular ligand in albumin and come in direct contact with the ligand, are presented in space-filling mode, with their hydrophobic and polar fragments differentiated by colors (Figure 6).

**Figure 7:**
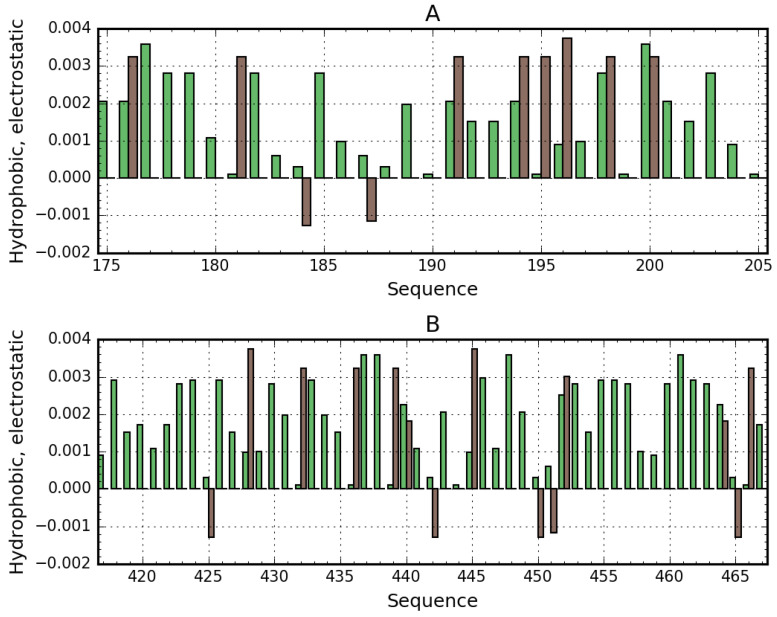
Electrostatic (brown) and hydrophobic (green) interactions in helices at 175–215 (**A**) and 417–467 (**B**). Bars represent charge and distinguish positively and negatively charged residues.

As suggested by our analysis, the site with potentially the highest capability to bind a suitably large ligand is the space between the helix at 175–215 and the one at 417–467, with calculated RD values of 0.664 and 0.534 respectively. Figure 7 provides a visualization of the theoretical (T) and observed (O) distribution profiles, highlighting the differences between the two. As predicted by the fuzzy oil drop model, this status indicates the ability to attract a large ligand—such as supramolecular Congo red (as well as the Congo red + Dox co-micelle). The relative instability of this region has been confirmed experimentally, with two distinct conformational patterns observed in albumin, denoted as N (native) and F structural form. F-form (classification according to [3] (p. 56)) is the albumin with parts A and B of the chain separated, keeping the rest of the chain folded in N form. In the latter conformation, the outlying domains (AI, AII and BIII) are differently oriented, extending the distance between the “arms” and the core.

To summarize, the presented cavity may be suspected of participation in binding large ligands for the following reasons:

1. its capacity for conformational rearrangements and therefore for accommodating a supramolecular ligand in its form of co-micelle (Congo red + Dox),
2. markedly increased hydrophobicity,
3. high positive electrostatic charge density (which promotes the binding of a negatively charged ligand—Figure 7).

Modeling results remain in agreement with the outcomes of experiments based on electrophoresis, gel filtration, and chromatography [12]. Albumin is capable of binding both Congo red and large co-micelles consisting of Congo red with intercalated doxorubicin [13].

### 3.4. Light Chain (Lambda) of IgG in the Fuzzy Oil Drop Classification 

Table 3 characterizes the status of a complex consisting of two IgG light chains, of individual chains as well as of individual domains. In each case, the status of the polypeptide chain is presented in the context of larger structural units (complex, chain, domain) as well as for standalone structures (with the encapsulating Gaussian adjusted for the chain being analyzed). We have also singled out beta strands and beta sheets comprising the beta sandwich structure, which typifies immunoglobulins.

**Figure 8:**
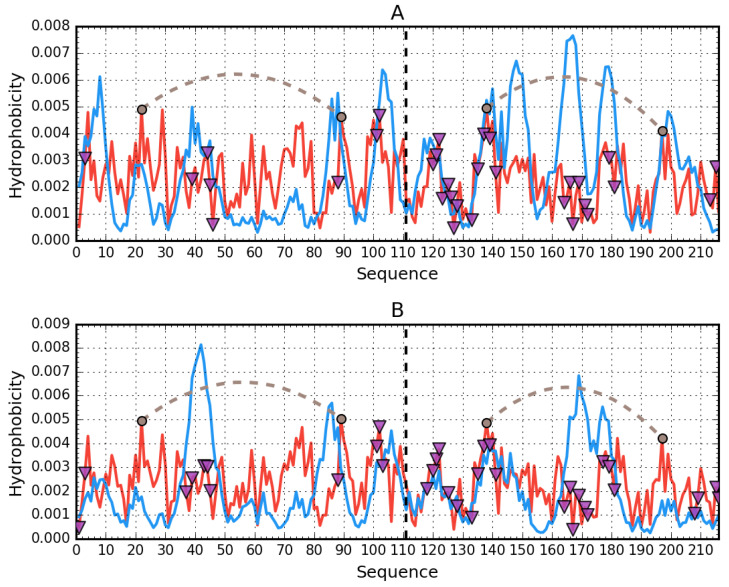
Hydrophobicity density distributions calculated for the whole light lambda chain of IgG as found in Bence-Jones complex (both chains treated as structural unit for 3D Gauss function): (**A**)—chain A; (**B**)—chain B. Blue lines—theoretical (T) distribution; red lines—observed (O) distribution; brown dashed curves—disulfide bonds; vertical dashed lines—domain boundaries; triangle markers—protein–protein interface in the complex.

The structure of the human IgG light chain dimer does not appear to include a common hydrophobic core. Likewise, each of its components (domains, complexation interfaces, and fragments not involved in complexation) appears to lack such a core. This is further highlighted by analysis of the dimer’s 3D structure (Figure 8).

Interpretation of results provided in Table 4 reveals significant differences with regard to the structure of each domain. VL domains do not conform to the monocentric distribution of hydrophobicity, while CL domains are quite accordant, with their observed distribution (O) closely in line with its theoretical counterpart (T). This suggests that CL domains are relatively more stable than VL domains.

In light of the results shown in Table 5, the CL-CL dimer appears to contain a hydrophobic core, which contributes to its structural stability. On the other hand, the VL dimer lacks such a core. The status of each beta sheet remains consistent with the status of its parent dimer.

Figure 8 provides a visualization of the results listed in Table 5, revealing the lack of a hydrophobic core—notably, local maxima predicted by T are not replicated in the observed distribution (O). Several fragments exhibit higher than expected hydrophobicity. From the shape of profiles shown in Figure 8 and values of RD (Table 5), we may also conclude that the IgG light chain dimer is highly asymmetrical. The profiles representing distribution of hydrophobic, electrostatic, and vdW interactions as identified in VL domain of human IgG are presented in Figure S7.

These conclusions, based on the interpretation of RD values, are borne out by analysis of T and O profiles for the presented structural units. In each case, the CL are regarded as more stable due to the presence of a hydrophobic core.

**Figure 9:**
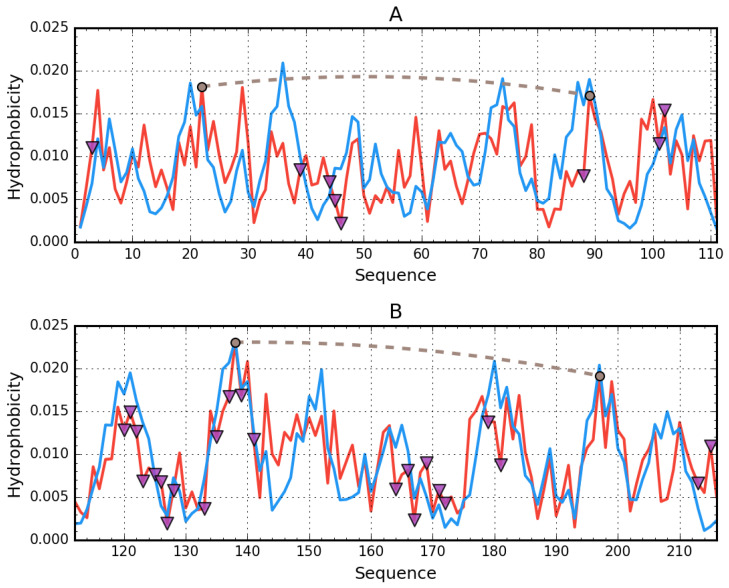
Hydrophobicity density distributions calculated for individual VL and CL as individual structural units: (**A**)—VL; (**B**)—CL. Blue lines—theoretical (T) distribution; red lines—observed (O) distribution; brown dashed curves—disulfide bonds; triangle markers—protein-protein interface in the complex.

Figure 9 reveals differences in the status of VL and CL domains (analyzed individually), with the profile of the VL domain providing a much closer match for theoretical values than in the case of the CL domain.

The status of fragments bracketed by disulfide bonds suggests strong stabilization of CL domains, contrary to VL domains.

Structural stability can also be assessed by looking at the interface area. Low values of RD for residues comprising the interface as well as for the remainder of the domain suggest good agreement with the monocentric hydrophobic core pattern. The same cannot be said for the VL domains, where the fragments separating disulfide bonds as well as the interface area, all deviate strongly from the theoretical distribution. A detailed discussion regarding the role of disulfide bonds can be found [14]. In the presented case, we can conclude that the CL domain structure appears consistent with the monocentric core model, while the VL dimer diverges from it.

The above analysis reflects the structural properties of IgG lambda chain dimers in the presented Bence-Jones protein. These consist of two individual (VL) domains, each of which is contributed to by a different chain. Similarly, two distinct CL domains pair up to form common structural units, and therefore it makes sense to analyze these dimers as a whole.

**Figure 10:**
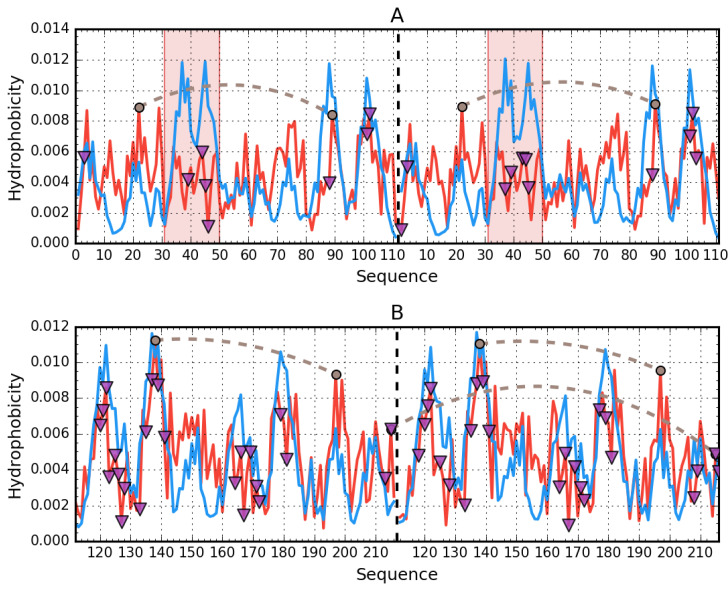
Hydrophobicity density distributions calculated for individual VL-VL and CL-CL domain dimers of IgG: (**A**)—VL-VL; (**B**)—CL-CL. Blue lines—theoretical (T) distribution; red lines—observed (O) distribution; brown dashed curves—disulfide bonds; vertical dashed—chain boundaries; triangle markers—protein–protein interface in the complex. Red background marks the location of disordered fragments (residues 31–50).

Comparative analysis of profiles shown in Figure 10A indicates a greater degree of similarity between T and O for the CL dimer than for the VL dimer. Likewise, the status of the interface area differentiates VL-VL and CL-CL, as listed in Table 5.

**Figure 11:**
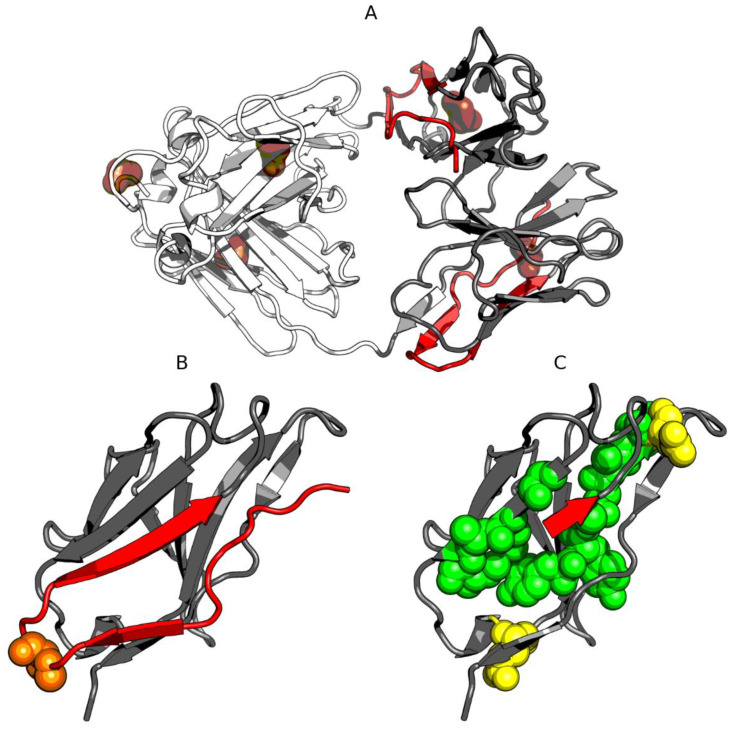
3D presentation of the light chain of IgG: (**A**)—whole complex (CL domain dimer—white; VL domain dimer—gray; red—1-23 fragment; brown spheres—disulfide bonds); (**B**)—VL domain (red—1–23 fragment; orange spheres—P19K mutation [16]); (**C**)—VL domain without the 1–20 fragment (red—21–23 fragment; green spheres—hydrophobic residues; yellow spheres—polar residues). Polypeptide chains that directly interact with Congo red are presented using the space-filling model.

Analysis of the dimer as a whole points to the V domain as the likely binding site for a large ligand such as supramolecular Congo red and its co-micelle form with Dox in particular. High RD values for beta sheets comprising the V domain suggest the capacity for structural rearrangements, including exposure of such sheets, for which the supramolecular ligand appears to exhibit conformational compatibility (the separation between individual Congo red molecules approximately matches the periodicity of peptide bonds in beta strands) [15]. Of note is residue 19 [16], where P19K mutation causes a significant loosening of the 1–22 loop (see the surface presentation in Figure 11), thereby introducing instability in the 1–20 fragment (marked in red).

In addition, the relatively low stability of the 23–67 fragment (distinguished—pink), as evidenced by the profiles plotted in Figure 10A, confirms the overall poor stability of the V domain. These results suggests that the V domain may be capable of significant structural rearrangements implicated in the complexation of such a large ligand as supramolecular Congo red (although its stability to bind supramolecular Congo red with intercalated Dox remains a mystery).

Results of experiments based on electrophoresis, gel filtration, and chromatography indicate formation of complexes consisting of CR and the light chain. On the other hand, the Congo red—Doxorubicin co-micelle does not appear to bind to the light chain under experimental conditions, and any putative complex between the co-micelle and the protein is so unstable that the two components migrate separately under electrophoresis [13].

### 3.5. Analysis of Pores, Clefts and Tunnels in the IgG Light Chain Dimer and in Albumin

Table 6 summarizes the sizes of the clefts, tunnels and pores present in the analyzed proteins.

Analysis of the geometrical properties of clefts, tunnels and pores (Table 6) indicates that their sizes are much greater in albumin. No tunnels have been identified in the Bence–Jones dimer. 

The sizes of clefts and tunnels—particularly the larger ones—suggest the ability to bind a supramolecular ligand. Additionally, existing clefts, tunnels, and pores may be susceptible to structural changes leading to enlargement due to the lack of a hydrophobic core in their corresponding domains.

## 4. Discussion

Supramolecular systems, in particular those which form ribbon-like micellar structures, were proposed to be used as carriers of drugs directed to selected targets marked by antibodies forming immune complexes [18,19,20]. Congo red typically represents such systems. Its micellar structure may attach many organic compounds including drugs by intercalation.

As, however, these supramolecular structures are stabilized by non-covalent bonds and thus may be destroyed by dilution, serum albumin which appears capable to incorporate particles of micellar material as ligands was used in these studies as a co-transporter.

This work was designed to localize binding sites in proteins engaged in the process. Fuzzy oil drop model, which allows theoretical prediction of optimal distribution of polarity in protein molecules and compares it to hydrophobicity distribution actually observed, was used here to localize the dye binding sites. However, according to used analysis, albumin domains fit relatively well to expectations, while the complete molecule does not. According to predictions by the model, much higher hydrophobicity is expected in the area surrounded by amino acids distinguished in Figure 4, where hydrophobic nucleus for the whole molecule is predicted by calculation. It exactly differentiates the cleft between two pseudo-symmetric units of albumin, which is an indication it is a special area. The large hydrophobic ligand brought in fulfils the gap, making the molecule closer to unity, which is now treated as a compact molecule.

The negative charge introduced by Congo red meets the predominantly charged amino acids (Figure 7). It hence confirms that cleft between pseudo-symmetric parts of albumin is the locus binding Congo red.

Albumin, which is a universal acceptor of drugs and dyes, does not require any prior structural modification and can form complexes with large numbers of Congo red molecules, with or without intercalated Dox; unlike the IgG light chain, where complexation is hampered by the relatively low size of the binding site, limiting interactions to just a handful of dye molecules. Free Dox is a positively charged compound, which does not bind to albumin; while the Congo red/Dox complex does. This opens up new possibilities regarding control of the dissemination of this toxic drug throughout the organism.

The presented analysis concerning immunoglobulins was carried out for the IgG light chain, which, following partial destabilization through heating, was used as a model of antibody-antigen immune complexes.

As remarked above, the heat-modified IgG light chain reflects the structural changes which, under natural conditions, are triggered by strain associated with formation of an immune complex, where bivalent antibodies must align to randomly distributed antigen determinants. The light chain/Congo red complex, which forms under elevated temperatures, has been subjected to electrophoretic analysis, revealing limited involvement of Congo red molecules (between 4 and 8 per light chain) [1,21,22,23,24,25,26].

Due to this limitation, the light chain remains incapable of binding the Congo red-Dox co-micelle: strong association of Congo red molecules around the positively charged Dox molecule results in a structure that is too large for the complexation site offered by the light chain. Furthermore, electrophoretic analysis provides no evidence of the putative capacity to bind additional Congo red molecules attached to those which are already bound by the protein, propagating outwards in a supramolecular fashion. Even if such structures form in the first step, electrophoretic migration destroys them. On the other hand, an immune complex, which consists of tightly packed molecules, appears to have greater capacity—as a scaffolding system—to attract supramolecular ligands in high amount. This is evidenced by participation of Dox in immune complexes formed in the presence of Congo red-Dox co-micelles, as proven by independent studies. Notably, elevated concentrations of Congo red are co-located with immune complexes in tissues analyzed in vivo by measuring the intensity of the characteristic color of the dye [2]. It is much higher than that attached directly to antibody molecules. In such a case, the greater quantity of Congo red anchored in between antibodies may intercalate various substances, as proven by the fluorizing agglutinates of erythrocytes formed in the presence of Congo red and rhodamine B [20]. This hypothesis is also supported by the large quantities of Congo red associated usually with thermally aggregated immunoglobulins [21].

## 5. Conclusions

The fuzzy oil drop model, applied in the presented study, enables us to detect binding sites for supramolecular ligands in albumin (particularly between its pseudo-symmetrical fragments) as well as in V domains, where complexation of dye molecules gives rise to a stable supramolecular structure, anchored in-between antibodies that participate in the immune complex.

## Figures and Tables

**Table 1:** Brief characteristics of proteins, which represent the focus of the presented study.

PDB- ID	Name	Source		Reference
1HK4	Albumin	HUMAN		[4]
4BJL	Light chain of IgG	HUMAN	Bence-Jones dimer	[5]

**Table 2:** Values of RD for various structural units: the complete albumin molecule and its individual domains. H—hydrophobic interactions; Ele—electrostatic interactions; vdW—van der Waals interactions.

Protein	Fragment	RD					
		H	vdW	Ele			
**Albumin** **(1HK4)**	3–584	**0.741**	**0.853**	**0.917**			
**Domain Status**							
**Domain**		**Domains in Molecule**			**Individual Domains**		
		**H**	**vdW**	**Ele**	**H**	**vdW**	**Ele**
AI	5–107	**0.692**	**0.843**	**0.860**	**0.547**	**0.764**	**0.834**
AII	108–197	**0.678**	**0.759**	**0.853**	**0.558**	**0.738**	**0.875**
AIII	205–296	**0.503**	**0.745**	**0.752**	0.434	**0.746**	**0.795**
BI	297–382	**0.718**	**0.820**	**0.908**	**0.542**	**0.665**	**0.847**
BII	383–494	**0.504**	**0.730**	**0.906**	0.461	**0.786**	**0.936**
BIII	495–570	**0.723**	**0.864**	**0.958**	0.465	**0.788**	**0.942**

**Table 3:** RD values calculated for various structural units: entire complex, individual domains, and VL-VL and CL-CL domain components of the complex. The table also lists the status of fragments bracketed by Cys residues, which participate in disulfide bonds. “P-P” corresponds to the status of the inter-chain interface, while “NO P-P” indicates the remainder of the chain, with interface residues excluded.

	Fragment	RD for Complex
DIMER	TWO CHAINS	**0.729**
INDIVIDUAL	DOMAIN VL	**0.737/0.611**
INDIVIDUAL	DOMAIN CL	**0.768/0.711**
	CHAIN A	**0.709**
	CHAIN B	**0.740**
SS bonds	22—89	**0.731/0.816**
	138—197	**0.607/0.736**
P-P		**0.643**
NO P-P		**0.724**

**Table 4:** Status of individual domains, along with their brief characteristics. “P-P” corresponds to the interface area; “NO P-P” indicates the remainder of the domain, with interface residues excluded. The table also lists fragments bracketed by Cys residues, which participate in SS bonds, as well as specific secondary folds. The asterisk “*”—the positions of edge-starting Beta-strands for particular Beta-sheets are given.

RD for Domains Treated as Individual Units		
	Fragment	RD
VL	1–111	**0.573/0.582**
P-P		**0.515/0.722**
NO P-P		**0.576/0.572**
SS	22–89	**0.541/0.544**
β-sheet	8–2 *	**0.611/0.585**
	18–22 *	0.402/0.425
CL	112–216	0.424/0.356
P-P		**0.505**/0.377
NO P-P		0.391/0.338
SS	138–197	0.376/0.343
β-sheet	118–122 *	0.492/**0.520**
	147–155 *	0.470/**0.502**

**Table 5:** RD values illustrating the status of complexes on the domain level. In each case, the 3D Gaussian is constructed in such a way as to encapsulate the whole dimer (VL or CL, as appropriate). The table also presents the status of the interface (P-P), of the remainder of the chain following exclusion of interface residues (NO P-P), of fragments bracketed by Cys residues participating in disulfide bonds, as well as for selected secondary and supersecondary folds. The asterisk “*”—the positions of edge-starting Beta-strands for particular Beta-sheets are given.

	RD Values for Dimers VL-VL CL-CL	
Domains	Fragments	RD
VL-VL	(1 − 111) + (1 − 111)	**0.716**
CL-CL	(112 − 216) + (112 − 216)	0.495
P-P		**0.599/0.366**
NO P-P		**0.695**/0.438
SS	22–89	**0.713/0.702**
	138–197	0.493/0.485
β-sheet	8–12 *	**0.778/0.789**
	18–22 *	0.355/0.444
	118–122 *	**0.559/0.547**
	147–155 *	**0.564/0.543**

**Table 6:** Sizes of clefts, tunnels and pores in the analyzed structures: IgG light chain dimer (Bence Jones complex—4BJL) and albumin (1HK4). Radius—radius of bottleneck; Free Radius—free radius of the bottleneck calculated without side chain atoms (flexibility of side chain). The “*” (asterisk) indicate that the ligand influences the analyzed structure. All data are derived from PDBSUM [4,5,17].

Clefts Volume Å^3^		Tunnels Radius Å/Free Radius Å		Pores Radius Å/Free Radius Å/Length Å	
**4BJL**	**1HK4**	**4BJL**	1HK4	4BJL	1HK4
11,137.50 1591.73 951.33 604.12 729.84 512.58 429.05 340.03 340.03 341.72	1713.78 * 2131.31 * 2035.55 * 1690.88 1125.98 1002.80 600.33 530.30 529.03 810.42	1.16/21.1 1.39/16.1 1.45/16.9 1.40/17.9 1.31/20.2	1.26/28.8 * 1.29/29.5 * 1.25/38.4 * 1.29/39.3 * 1.25/39.3 * 1.30/40.1 * 1.26/48.9 * 1.31/49.7 *	ABSENT	/3.58/27.8 1.16/1.17/37.0 1.57/3.20/42.9 1.97/4.70/48.3 1.96/4.72/55.2 * 1.88/2.88/58.6 * 1.17/1.21/98.6 * 2.09/2.10/98.0 *

## Data Availability

The data can be available on request addressed to corresponding author.

## Supplementary Materials

The following are available online at https://www.mdpi.com/article/10.3390/biom11040501/s1, Figure S1: Profiles: T—red, R—blue and O—green for domain 1. —distribution represents: A—hydrophobic, B—electrostatic and C—vdW interaction, Figure S2. Profiles: T—red, R—blue and O—green for domain 2. O—distribution represents: A—hydrophobic, B—electrostatic and C—vdW interaction, Figure S3. Profiles: T—red, R—blue and O—green for domain 3. O—distribution represents: A—hydrophobic, B—electrostatic and C—vdW interaction, Figure S4. Profiles: T—red, R—blue and O—green for domain 4. O—distribution represents: A—hydrophobic, B—electrostatic and C—vdW interaction, Figure S5. Profiles: T—red, R—blue and O—green for domain 5. O—distribution represents: A—hydrophobic, B—electrostatic and C—vdW interaction, Figure S6. Profiles: T—red, R—blue and O—green for domain 6. O—distribution represents: A—hydrophobic, B—electrostatic and C—vdW interaction, Table S1. Status of domains in albumin. Domains are treated as individual structural units in this calculation. RD values as described in Materials and Methods: H—hydrophobic, Ele- electrostatic, vdW—van der Waals interactions respectively, Table S2. RD values expressing the status of polypeptide chain fragments determine by the positioned of halfCys in albumin treated as structural unit and in domains treated as individual structural units, Table S3. Status of residues engaged in ligand complexation (Ligand) and residues not-engaged in ligand binding (No-Lig).Bold values distinguish the positions with RD > 0.5. Calculation performed for H interactions, Table S4. Status (H-interaction) of helical fragments in complete molecule as well in domains treated as individual structural units, Figure S7. Status of VL domain of IgG expressed by profiles T—red, O—green and R—blue for interaction: A—hydrophobic, B—electrostatic and C—vdW interaction.
