# Supplementary material for: Structure and Location of Protein Sites Binding Self-Associated Congo Red Molecules with Intercalated Drugs as Compact Ligands—Theoretical Studies
Source: Biomolecules. 2021 Mar 26;11(4):501. doi: 10.3390/biom11040501 (PMC8065709; doi:10.3390/biom11040501)
Supplement: Supplementary file 1 [file biomolecules-11-00501-s001.pdf]

biomolecules

Supplementary Materials

Structure and Location of Protein Sites Binding Self-AssociatedCongo Red Molecules with Intercalated Drugs as Compact-Ligands—Theoretical Studies

Ptak-Kaczor Magdalena [1], Kwiecińska Klaudia [2], Korchowiec Jacek [2], Chłopaś Katarzyna [3], Banach Mateusz [1], Roterman Irena [1] and Jagusiak Anna [3,*]

[1] Department of Bioinformatics and Telemedicine, Faculty of Medicine, Jagiellonian University MedicalCollege, Medyczna 7, 30-688 Krakow, Poland; magdalena.ptak@uj.edu.pl (P.-K.M.);mateusz.banach@uj.edu.pl (B.M.); irena.roterman-konieczna@uj.edu.pl (R.I.)
[2] Department of Theoretical Chemistry, Faculty of Chemistry, Jagiellonian University, K. Gumi ́nski,Gronostajowa 2, 30-387 Kraków, Poland; klaudia.kwiecinska@student.uj.edu.pl (K.K.);korchow@chemia.uj.edu.pl (K.J.)
[3] Faculty of Medicine, Chair of Medical Biochemistry, Jagiellonian University Medical College, Kopernika 7,31-034 Krakow, Poland; kchlopas@su.krakow.pl
* Correspondence: anna.jagusiak@uj.edu.pl; Tel.: +48-12-42-274-00

The detailed analysis of albumin and VL domain of human IgG is presented in this part of the paper. Results present the characteristics of discussed proteins and domains taking the fuzzy oil drop model to analyse the status of molecules and their domains in context of possible large-size supramolecular ligand binding.

A

B

C

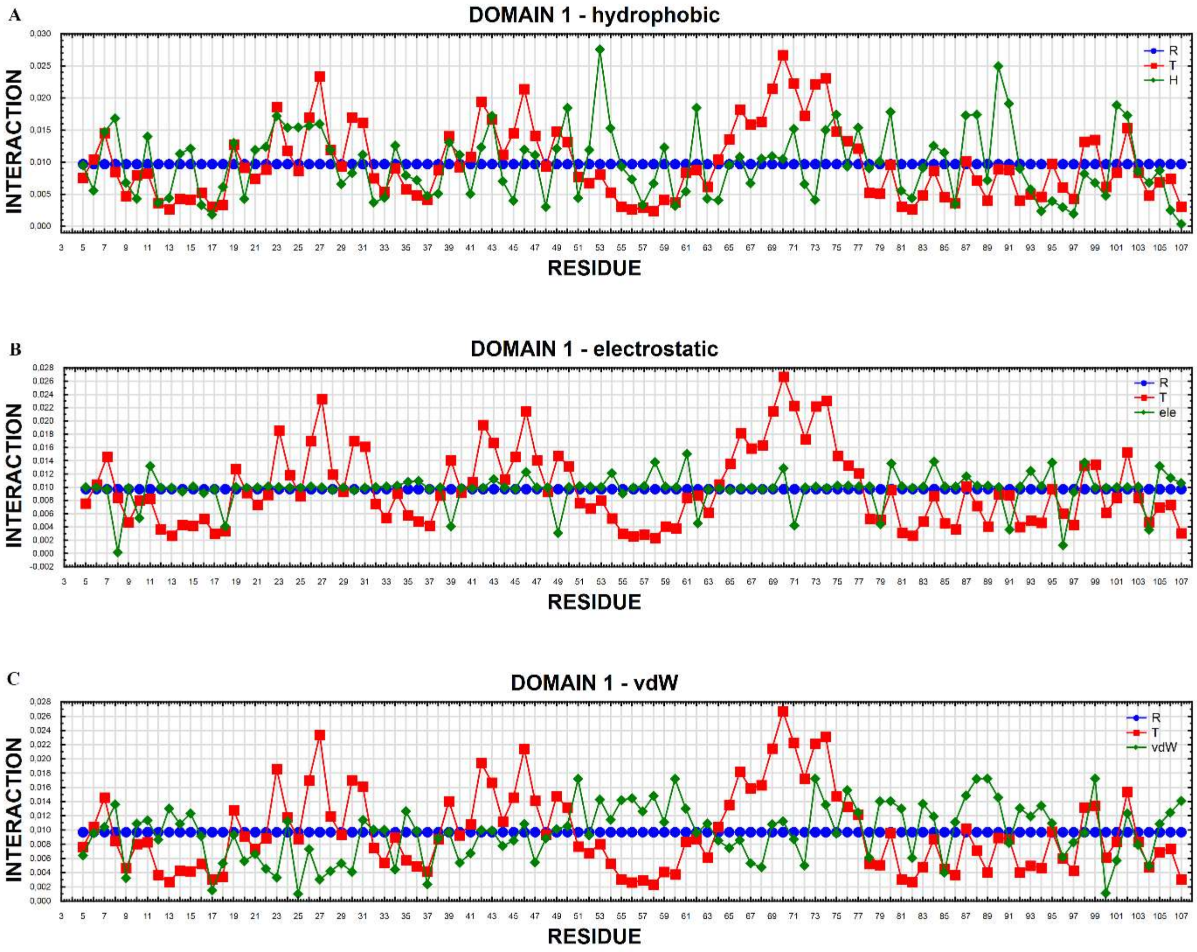

Figure S1. Profiles : T - red, R - blue and O - green for domain 1. O - distribution represents: A - hydrophobic, B - electrostatic and C - vdW interaction.

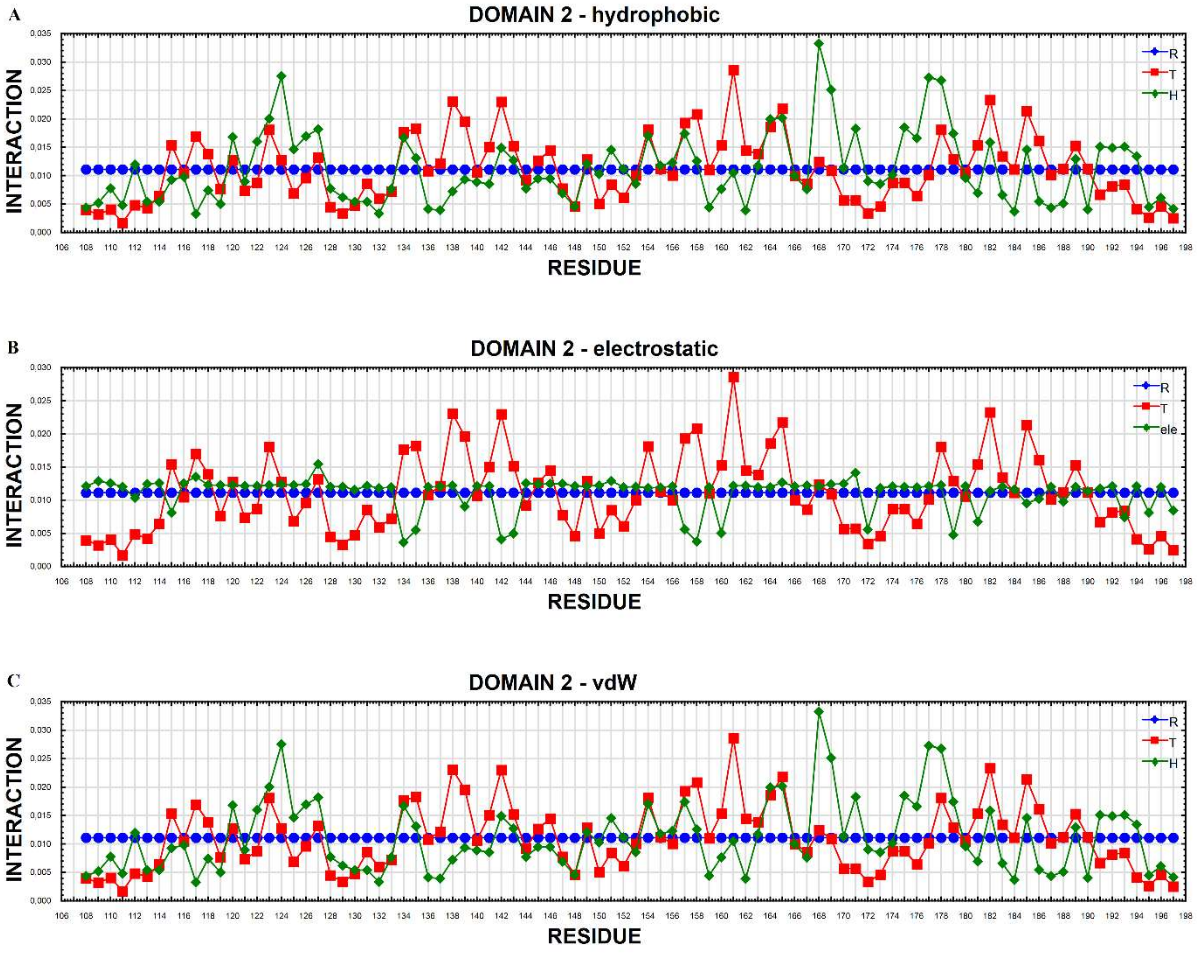

Figure S2. Profiles : T - red, R - blue and O - green for domain 2. O - distribution represents: A - hydrophobic, B - electrostatic and C - vdW interaction.

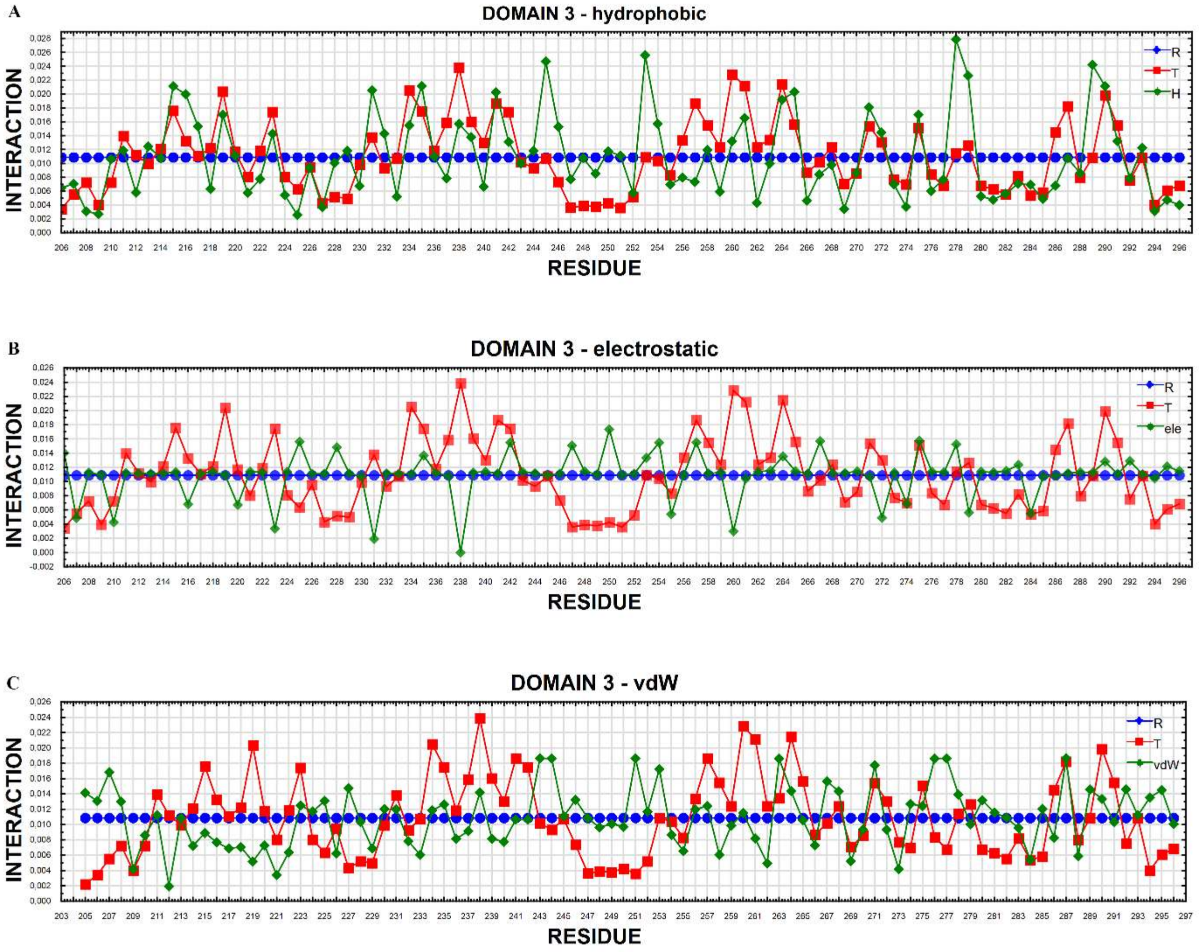

Figure S3. Profiles : T - red, R - blue and O - green for domain 3. O - distribution represents: A - hydrophobic, B - electrostatic and C - vdW interaction.

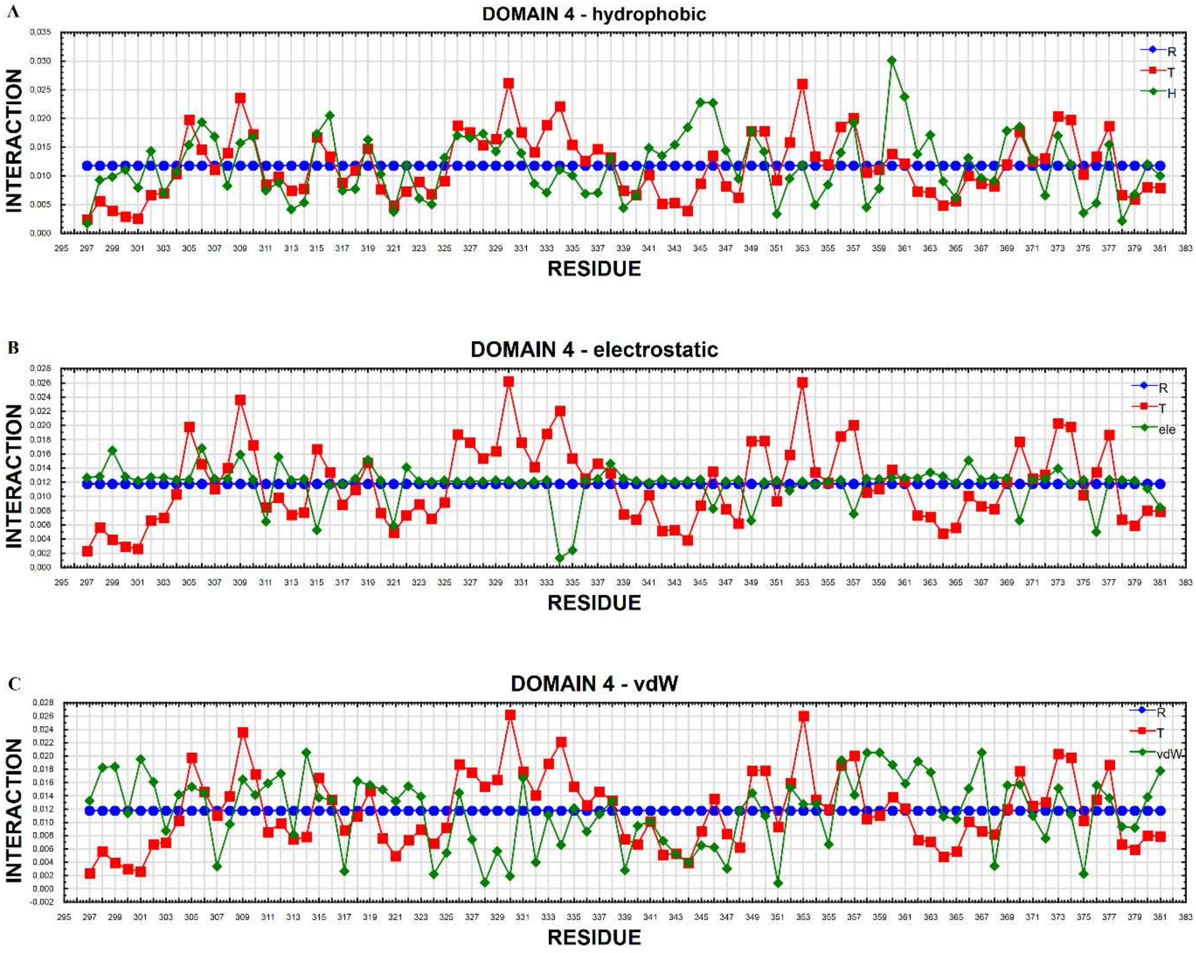

Figure S4. Profiles : T - red, R - blue and O - green for domain 4. O - distribution represents: A - hydrophobic, B - electrostatic and C - vdW interaction.

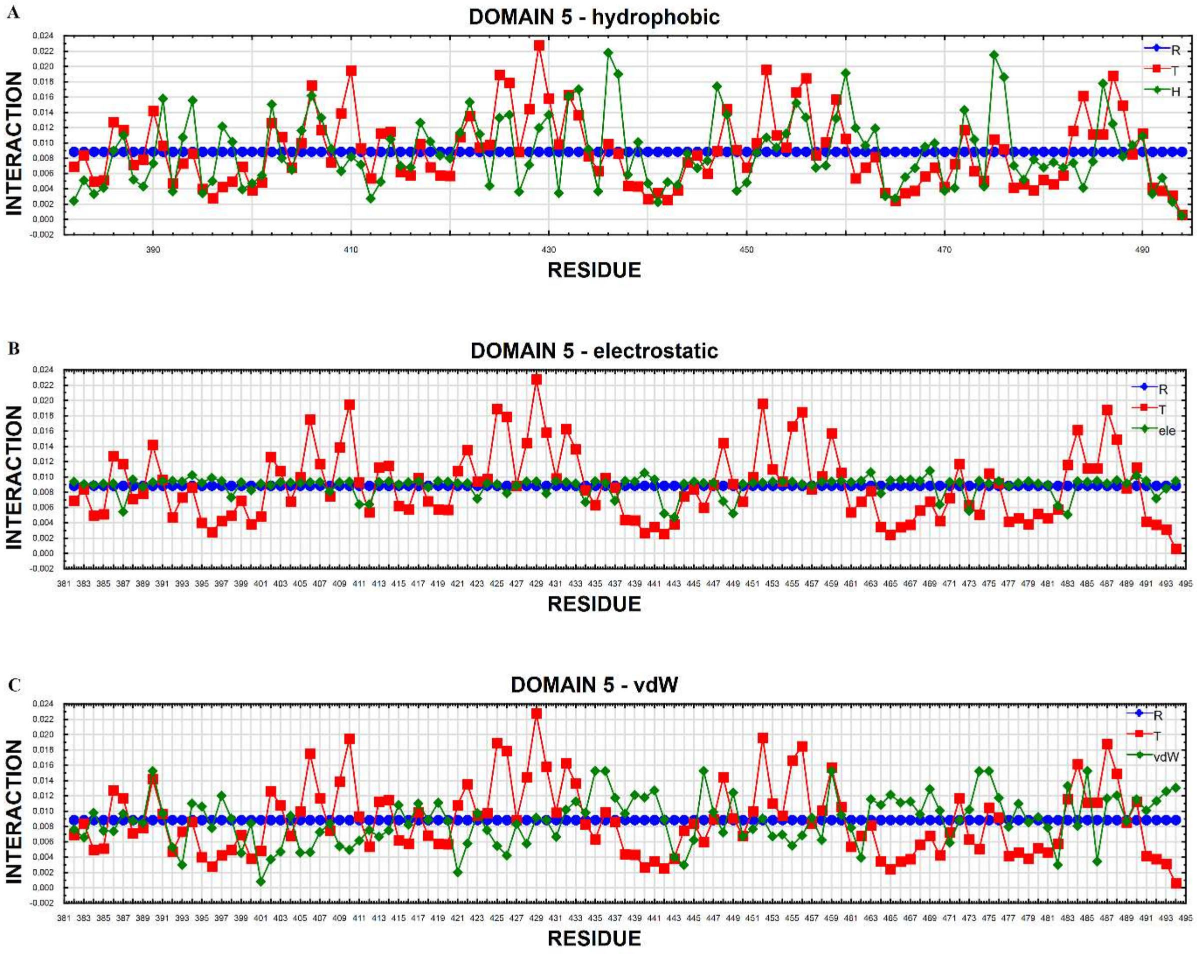

Figure S5. Profiles : T - red, R - blue and O - green for domain 5. O - distribution represents: A - hydrophobic, B - electrostatic and C - vdW interaction.

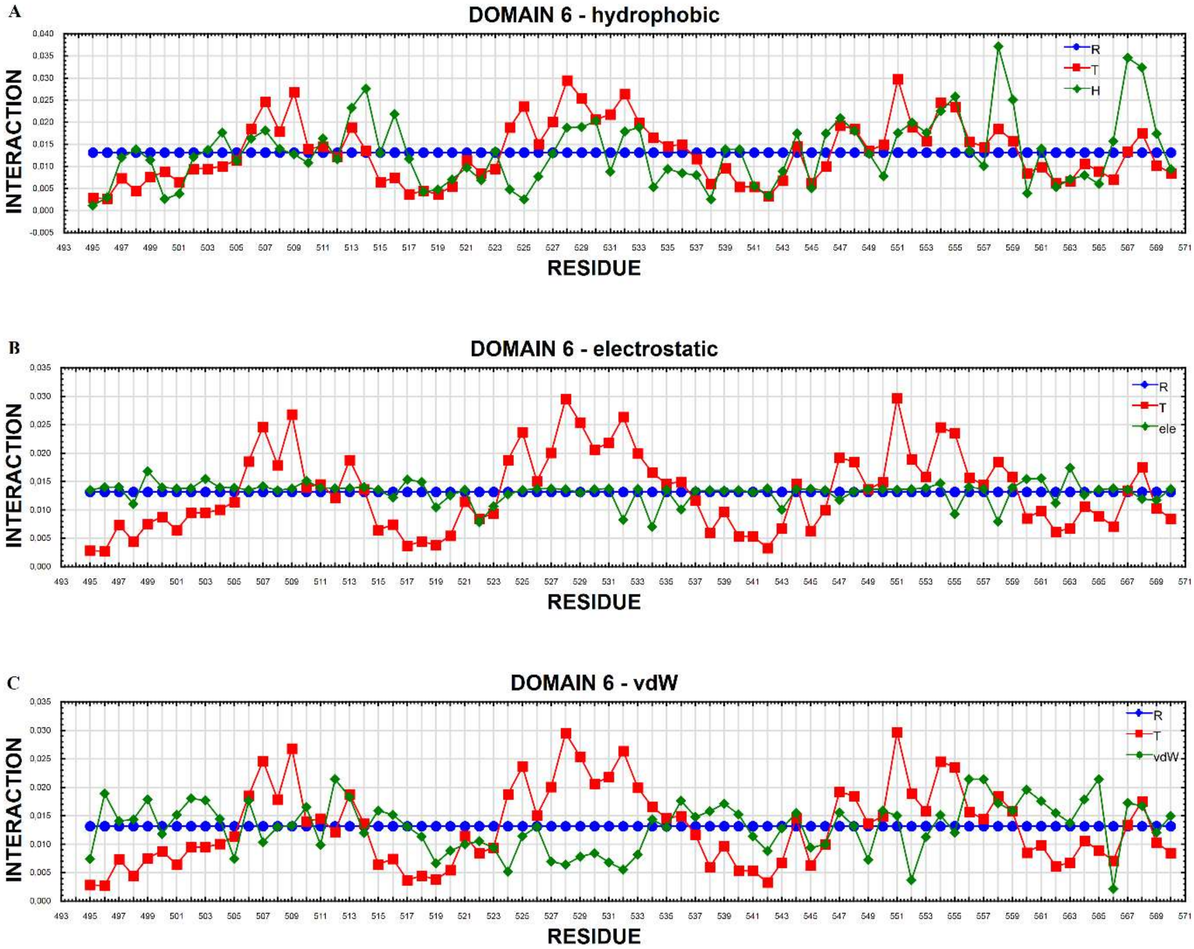

Figure S6. Profiles : T - red, R - blue and O - green for domain 6. O - distribution represents: A - hydrophobic, B - electrostatic and C - vdW interaction.

Non-bonding interaction in albumin - evaluation on the basis of fuzzy oil drop model

To assess the status of the proteins the identification of the distribution of other than hydrophobic interaction is necessary. Calculation revealing the distribution of vdW and electrostatic interaction can show whether the concentration of these interaction is also of 3D Gauss category. Similarly to hydrophobic interaction characteristics the 3D Gauss function (T) as well as unified distributions are taken as reference distributions for divergence entropy calculation. Status of each residue is expressed in O distribution as the sum of interaction of particular residue with all others present in the molecule.

Values of RD given in Table S1 reveal high similarity of observed distribution versus R distribution. It means that the electrostatic interaction does not generate any high concentration in central part of the molecule. The vdW interactions are characterised by similar status.

Table S1. Status of domains in albumin. Domains are treated as individual structural units in this calculation. RD values as described in Materials and Methods: H - hydrophobic, Ele- electrostatic, vdW - van der Waals interactions respectively.

DOMAIN	RD for INTERACTIONS		
	**vdW**	**Ele**	**H**
AI	**0.764**	**0.834**	**0.546**
AII	**0.738**	**0.875**	**0.558**
AIII	**0.746**	**0.795**	0.434
BI	**0.665**	**0.847**	**0.550**
BII	**0.786**	**0.936**	0.448
BIII	**0.788**	**0.942**	0.464

Status of polypeptide chain fragments determined by SS-bonds

The hydrophobic interaction in form of hydrophobic core together with the SS-bonds system in protein molecule are the factors responsible for tertiary structure stabilisation. This is why it is interesting to see whether these two factors collaborate in this purpose.

As it can be seen in Table S2 status of polypeptide chain fragments between two halfCys positions generating SS-bond is accordant with 3D Gauss distribution for H interaction in all cases when RD < 0.5.

Status described by RD > 0.5 suggests possibile elasticity for certain polypeptide chain fragment. Values of RD in Table S2 reveals differentiate status of certain polypeptide chain fragments.

Table S2. RD values expressing the status of polypeptide chain fragments determine by the positioned of halfCys in albumin treated as structural unit and in domains treated as individual structural units.

RD for polypeptide chain fragments determined by the positions of halfCys in complete molecule and in domains			
**DOMAIN**		**IN MOLECULE**	**IN INDIVIDUAL DOMAINS**
AI	53–62	0.319	0.372
	75–91	0.434	0.356
	90–101	**0.614**	0.466
AII	124–169	**0.628**	**0.530**
	168–176	0.383	0.240
AIII	200–246	0.470	0.401
	245–253	0.314	0.172
	265–279	0.326	0.323
	278–289	**0.556**	0.406
BI	316–361	**0.640**	**0.578**
	360–369	0.161	0.256

BII	392–438	**0.545**	0.490
	437–448	0.361	0.249
	461–477	0.352	0.227
	476–487	**0.548**	**0.531**
BIII	514–559	**0.702**	0.489
	558–567	0.436	0.296

Status of residues engaged in ligand binding

Status of residues engaged in ligand binding represents local discordance with idealised hydrophobicity distribution. Usually it is expressed by local hydrophobicity deficiency. It is caused by the presence of local cavity ready for ligand complexation.

This is why the residues usually represent the status expressed by RD > 0.5 (columns - Ligand). Column describes as NO-Lig - RD values for residues not engaged in ligand binding.

Table S3. Status of residues engaged in ligand complexation (Ligand) and residues not-engaged in ligand binding (No-Lig).Bold values distinguish the positions with RD > 0.5. Calculation performed for H interactions.

RD for ligand binding residues in complete molecule and in domains treated as individual structural units

	COMPLETE MOLECULE	DOMAINS
LIGAND	**0.711**	
NO-LIG	**0.731**	
DOMAIN AI 5-107		
DOMAIN AII 108-197		
LIGAND	**0.638**	**0.644**
NO LIG	**0.660**	**0.527**
DOMAIN AIII 215-296		
LIGAND	**0.574**	0.412
NO LIG	**0.503**	0.433
DOMAIN BI 297-382		
LIGAND	0.374	**0.730**
NO LIG	**0.703**	**0.514**
DOMAIN BII 383-494		
LIGAND	**0.586**	0.449
NO LIG	0.470	0.402
DOMAIN BIII 495-570		
LIGAND	**0.709**	**0.578**
NO LIG	**0.741**	0.440

Status of BI domain is exceptional revealing low RD for residues engaged in ligand binding taking the complete molecule as the structural unit. While the status of ligand binding residues in individual domain is described by RD > 0.5.

Status of parts of domains AII and BI not engaged in ligand bind suggests the presence of other factors influence the structuralisation of these domains.

Status of helical fragments in albumin revealing the participation of helices in hydrophobic core formation

Albumin is characteristic by high presence of helical forms.

The question is : To what extend the participate in hydrophobic core formation ?

Their status in complete molecule and in individual domains can be treated as similar.

Less than half reveals the status of RD > 0.5. Such status may suggest the possible elasticity of certain polypeptide chain fragments

On the other hand this elasticity may be limited by the presence of SS-bonds. However the flexibility of helices may have important role in ligand binding particularly ligand of large size as it is discussed in this paper.

Table S4. Status (H-interaction) of helical fragments in complete molecule as well in domains treated as individual structural units.

RD values for helical fragments - complete molecule and domains taken as structural units

DOMAINS	FRAGMENT	COMPLETE MOLECULE	DOMAIN
AI	5–15	**0.620**	**0.518**
	16–31	0.415	0.385
	35–56	**0.617**	**0.605**
	67–75	**0.678**	**0.559**
	79–85	0.383	0.141
	86–93	0.440	0.205
	96–105	**0.506**	0.405
AII	119–130	0.416	0.259
	131–146	0.433	0.380
	150–169	**0.662**	**0.577**
	173–197	**0.702**	**0.571**
AIII	206–223	**0.512**	0.324
	227–248	0.451	**0.565**
	249–267	**0.502**	**0.589**
	268–271	0.190	0.213
	275–280	0.343	0.324
	285–293	**0.522**	0.450
BI	304–310	**0.655**	**0.609**
	314–321	0.461	0.211
	322–336	0.462	0.483
	342–359	0.464	**0.688**
	365–370	0.078	0.116
	372–382	0.499	0.368

BII	383–398	0.482	0.479
	399–415	0.468	0.469
	419–438	**0.596**	**0.512**
	441–467	0.429	0.389
	470–479	0.379	0.267
	483–490	**0.591**	**0.605**
BIII	504–508	**0.711**	**0.771**
	512–516	**0.534**	**0.579**
	517–536	**0.600**	**0.530**
	540–560	**0.563**	0.337
	565–570	0.457	0.295

The status of VL domain in respect to the hydrophobic, electrostatic and vdW interaction calculated on the basis of fuzzy oil drop model is resented on Figure S7.

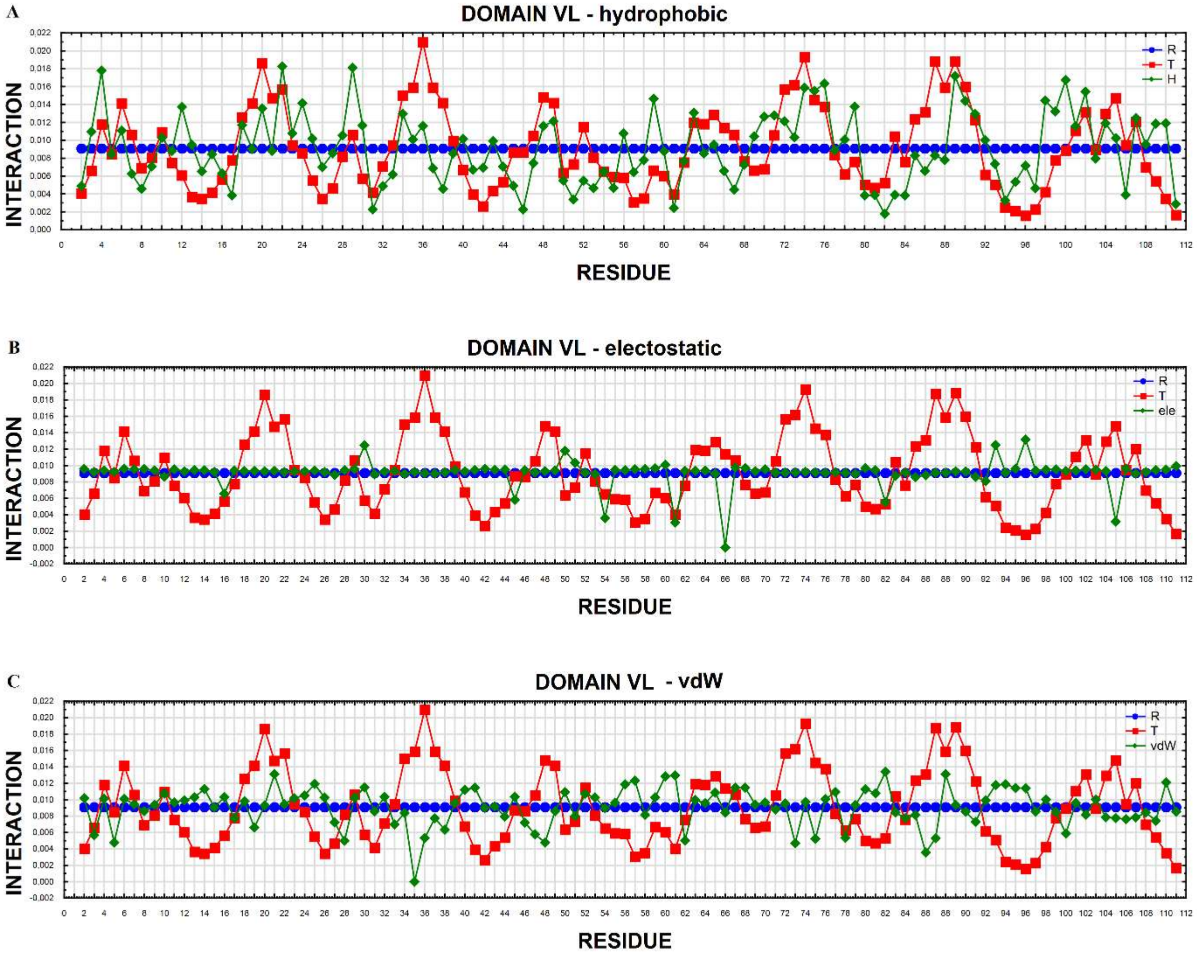

Figure S7. Status of VL domain of IgG expressed by profiles T - red, O - green and R - blue for interaction: A - hydrophobic, B - electrostatic and C - vdW interaction.
